# Aleutian mink disease virus in furbearing mammals in Nova Scotia, Canada

**DOI:** 10.1186/1751-0147-55-10

**Published:** 2013-02-08

**Authors:** A Hossain Farid

**Affiliations:** 1 Department of Plant and Animal Sciences, Faculty of Agriculture, Dalhousie University, Truro, Nova Scotia, B2N 5E3, Canada

**Keywords:** Aleutian mink disease virus, American mink, Carnivores, Furbearing species, *Mustelidae*, Rodents

## Abstract

**Background:**

Aleutian mink disease virus (AMDV) is widespread among ranched and free-ranging American mink in Canada, but there is no information on its prevalence in other wild animal species. This paper describes the prevalence of AMDV of 12 furbearing species in Nova Scotia (NS), Canada.

**Methods:**

Samples were collected from carcasses of 462 wild animals of 12 furbearing species, trapped in 10 NS counties between November 2009 and February 2011. Viral DNA was tested by PCR using two primer pairs, and anti-viral antibodies were tested by counterimmunoelectrophoresis (CIEP) on spleen homogenates.

**Results:**

Positive PCR or CIEP samples were detected in 56 of 60 (93.3%) American mink, 43 of 61 (70.5%) short-tailed weasels, 2 of 8 (25.0%) striped skunks, 2 of 11 (18.2%) North American river otters, 9 of 85 (10.6%) raccoons, and 2 of 20 (10.0%) bobcats. Samples from six fishers, 24 coyotes, 25 red foxes, 58 beavers, 45 red-squirrels and 59 muskrats were negative. Antibodies to AMDV were detected by CIEP in 16 of 56 (28.6%) mink and one of the 8 skunks (12.5%). Thirteen of the mink were positive for PCR and CIEP, but three mink and one skunk were CIEP positive and PCR negative. Positive CIEP or PCR animals were present in all nine counties from which mink or weasel samples were collected.

**Conclusions:**

The presence of AMDV in so many species across the province has important epidemiological ramifications and could pose a serious health problem for the captive mink, as well as for susceptible wildlife. The mechanism of virus transmission between wildlife and captive mink and the effects of AMDV exposure on the viability of the susceptible species deserve further investigation.

## Background

Free-ranging American mink populations are infected with the Aleutian mink disease virus (AMDV) across Canada [1-5] and in several European countries [6-9]. The occurrence of natural infection with or exposure to AMDV in a few members of the *Mustelidae* family (e.g., European mink, ferrets, polecats, stone martens, pine martens, Eurasian otters), and other carnivores (striped skunks, common genets, raccoons, foxes) has also been reported [6,8,10-14]. Information on the prevalence of AMDV in wildlife in Eastern Canada is limited to one report on the feral American mink [3]. The primary objective of this study was to survey the prevalence of AMDV in wild furbearing species in Nova Scotia (NS), the largest ranched mink pelt producing province in Canada. The use of spleen as a source of anti-AMDV antibodies and the utilization of two PCR primer pairs, to improve the likelihood of detecting exposure to AMDV in animal cadavers, were also investigated.

## Methods

### Animal sampling

**Figure 1:**
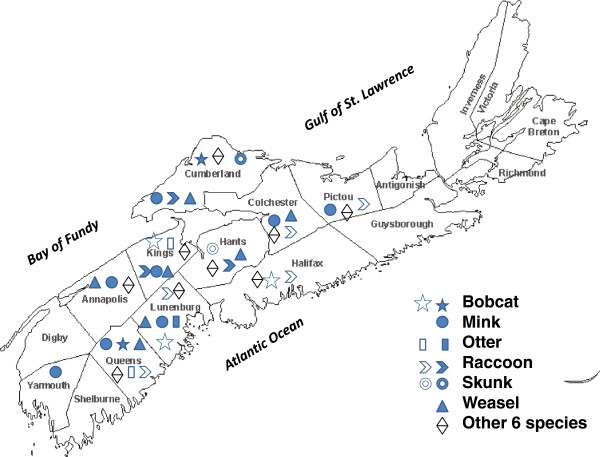
**Geographical distribution of species tested in different counties of Nova Scotia.** Solid signs and open signs represent Aleutian mink disease virus infected and non-infected species, respectively.

Spleen samples from 462 animals, representing 12 furbearing species, were collected in 10 counties in NS between November 2009 and February 2011 (Figure 1). Samples were collected from Mustelids including American mink (*Neovison vison*), short-tailed weasel (*Mustela erminea*), fisher (*Martes pennanti*) and river otter (*Lontra canadensis*); carnivores, including coyote (*Canis latrans*), red fox (*Vulpes vulpes*), raccoon (*Procyon lotor*), striped skunk (*Mephitis mephitis*) and bobcat (*Lynx rufus*); and rodents, including muskrat (*Ondatra zibethicus*), beaver (*Castor canadensis*) and red squirrel (*Tamiasciurus hudsonicus*). Animals were captured by licensed trappers during the trapping season, in accordance with provincial regulations. For spleen collection, trappers were provided with sampling instructions and sufficient supplies (scalpel blades, disposable gloves, sample bags) to minimize the chance of cross contamination. The location where trapping occurred and the gender of some animals were recorded by the trappers on a data collection form that was designed for this survey. Most carcasses were kept frozen and spleen samples were collected after they were thawed for pelting. Samples were placed in plastic bags, identified and stored in house freezers (−15°C) before delivery to the laboratory on ice for long-term storage at −80°C. In addition, carcasses of four free-ranging mink from the province of New Brunswick were tested.

### Laboratory procedures

Cell-free homogenates were prepared from 0.25 g of spleen sample in 0.75 ml of sterile phosphate buffered saline (25% W/V) and centrifuged at 16,000 g for 10 min. DNA was extracted from 200 μL of cell-free supernatant, using the Dynabeads Silane viral NA kit, according to the manufacturer’s protocol (Invitrogen, Burlington, ON), and eluted in 100 μL elution buffer. DNA was also extracted from mink spleen tissues by the high-salt procedure [15] with the addition of an RNAse treatment, using 2 μL of a 10 μg/μL RNAse and incubated at 37°C for 30 min.

Several primer pairs were designed using the sequences of the AMDV from Genbank (http://www.ncbi.nlm.nih.gov/genbank) and strains that are circulating in NS (sampled by the author) using the Oligo Primer Analysis Software, Version 6 (Molecular Biology Insight, Cascade, CO, USA) in order to amplify the AMDV by the polymerase chain reaction (PCR). Two pairs of primers (P60 and P70) were used in this experiment. The primer sequences were 60F: 5’-GGGTGTATGGATGAGTCCTAAA, 60R: 5’-CCCCAAGCAACG TGTACT, 70F: 5’-ACGAGGTAG ACC TATTAG ATGG and 70R: 5’-GCATGTTAC TTGGCTTAGTTTG, corresponding to nucleotides 2771–2792, 3302–3285, 3646–3667 and 4208–4187, respectively, of the VP2 gene of the ADV-G (Genbank accession number NC_001662).

PCR amplifications were performed in 15 μL total volumes containing final concentrations 0.1% Tween 20, 1X PCR buffer, 0.2 mM each dNTPs (Roche, Mississauga, ON, Canada), 400 nM each primer, 0.8 unit of *Taq* polymerase (Invitrogen) and 2.5 mM MgCl_2_. Three PCR tests were carried out on each sample using 1.5, 2.5 and 3.5 μL of DNA. This battery of tests was repeated when there were faint or no amplifications. In cases where one faint band was observed in six runs, PCR tests were repeated for the third time (up to nine amplifications/ primer/ sample). A sample was declared PCR positive when at least two reactions from at least one of the primer pairs were successful. The sample was considered negative when no amplification occurred or when only one of the nine reactions produced a faint amplification. Mink DNA samples extracted by the high-salt procedure were amplified by the primer 60F/60R using four DNA volumes (1.7X, X, X/10, X/20, where X is 1.5 μL of the stock DNA in 15 μL final PCR reaction mixture). This panel was repeated as explained above.

The thermal cycler was programmed at 95°C initial denaturation for 5 min, followed by 30 cycles of 94°C denaturation, 56.4°C annealing and 72°C extension, each for 60 sec, with a final extension at 72°C for 6 min. A reaction containing DNA from a known AMDV-infected animal (positive control) and a reaction containing DNA from an AMDV-free mink (negative control) were included in all tests. PCR products were run on agarose gels, stained with ethidium bromide and visualized under UV light. To avoid contamination, sterile filter-tips were used, and sample preparation, DNA extraction, PCR cocktail preparation, PCR amplification and gel electrophoresis were performed in four different laboratories with unidirectional sample movement.

Counterimmunoelectrophoresis (CIEP) was carried out on duplicate 50 μl samples of cell-free supernatants by the Animal Health Laboratory of the NS Department of Agriculture in Truro, NS. The test was performed in agarose gels using an antigen produced by the Research Foundation of the Danish Fur Breeders Association.

### Data analysis

Data were analyzed using SAS, Version 9.2 [16]. The Likelihood Ratio Chi-square tests or Fisher’s Exact Tests, when applicable, were used to analyze the difference between the amplification success of the two primers, the differences between sexes and among counties for the AMDV prevalence in free-ranging mink, weasels and raccoons, as well as the difference between the amplification success of DNA extracted from mink by high-salt and cell-free media using 60F/60R primers. The agreements between the results of PCR tests by the two primers and DNA extraction methods were tested by the Kappa coefficient, which is expected to be a more robust measure of association than simple percent agreement because it takes into account the agreement that occurs by chance. Confidence intervals were computed by PROC SURVEYMEANS.

## Results

Spleen samples were collected by 17 trappers in NS, 12 of whom supplied samples from mink. The prevalence of AMDV exposure, measured as those that were CIEP or PCR positive, was the highest in American mink (56 of 60, 93.3%), followed by short-tailed weasels (43 of 61, 70.5%), striped skunks (2 of 8, 25.0%), North American river otters (2 of 11, 18.2%), raccoons (9 of 85, 10.6%) and bobcats (2 of 20, 10.0%). The virus was not detected in fishers, coyotes, red foxes, beavers, red squirrels or muskrats (Table 1). All four mink from New Brunswick were PCR positive. Of the animals whose gender was recorded, 8 of 56 mink (14.3%), 6 of 57 weasels (10.5%) and 26 of 79 raccoons (36.1%) were females. The prevalence of AMDV was not different between male (95.8%) and female (100%) mink (χ^2^ = 0.6, *P* = 0.73), male (68.6%) and female (66.7%) weasels (χ^2^ = 0.01, *P* = 0.92) or male (11.3%) and female (7.7%) raccoons (χ^2^ = 0.3, *P* = 0.61).

**Table 1:** **Summary of testing of wild furbearers for Aleutian mink disease virus by CIEP and PCR in Nova Scotia, Canada (2009–2011)**^a,b^

**Species**	**AN**	**CO**	**CU**	**HX**	**HA**	**KI**	**LU**	**PI**	**QU**	**YA**	**Mean and 95% CL**
Bob cat			1/5 (20.0)	0/1 (0.0)		0/1 (0.0)	0/4 (0.0)		1/9 (11.1)		2/20 (10.0)
											−4.4% - 24.4%
Coyote			11			2	8		3		0/24
Fisher		3	3								0/6
Fox		3	13		3	1	3		2		0/25
Mink	4/4^c^	7/7	13/14			9/11	18/19	2/2 ^d^	1/1	2/2	56/60 (93.3)
	(100)	(100)	(92.8)			(81/8)	(94.7)	(100)	(100)	(100)	86.8% - 99.9%
Otter						0/1 (0.0)	2/7 (28.6)		0/3 (0.0)		2/11 (18.2)
											−9.0%- 45.4%
Raccoon		0/4	2/26	0/7	1/17	6/10	0/16	0/4	0/1		9/85 (10.6)
		(0.0)	(7.7)	(0.0)	(5.9)	(60.0)	(0.0)	(0.0)	(0.0)		3.9%-17.3%
Skunk			2/6 ^e^ (33.3)		0/2 (0.0)						2/8 (25.0)
											−13.7% - 63.7%
Weasel	4/4 (100)	4/5 (80.0)	15/21 (71.4)		7/11 (63.6)	2/2 (100)	6/11 (54.6)		5/7 (71.4)		43/61 (70.5)
											58.7%-82.3%
Sub-total	8/8	11/22	33/99	0/8	7/33	17/28	26/68	2/6	7/26	2/2	114/300 (38.0)
	(100)	(50.0)	(33.3)	(0.0)	(21.2)	(60.7)	(38.2)	(33.3)	(26.9)	(100)	
Beaver	1	10	13	2	11	7	7	6	1		0/58
Muskrat		3	10		10	10	10	10	6		0/59
Squirrel			25		10		6	4			0/45
Sub-total	1	13	48	2	31	17	23	20	7	0	0/162
Total	9	35	147	10	64	45	91	26	33	2	462

^a^ Number of positive animals over number tested. Percentages of positive animals are shown in brackets.

PCR: Polymerase chain reaction.

CIEP: Counterimmunoelectrophoresis.

^b^ AN = Annapolis, CO = Colchester, CU = Cumberland, HX = Halifax, HA = Hants, KI = Kings, LU = Lunenburg, PI = Pictou, QU = Queens, YA = Yarmouth.

^c^ Two of the 4 mink were CIEP positive but PCR negative.

^d^ One of the two mink was CIEP positive but PCR negative.

^e^ One of the two skunks was CIEP positive but PCR negative.

### CIEP

Antibodies to AMDV were detected by CIEP in 16 of 56 mink (28.6%) and one of the 8 skunks (12.5%). Thirteen of the CIEP positive mink were also PCR positive, but three mink and one skunk were CIEP positive but PCR negative. Of the 56 mink whose genders were recorded, 13 (23.2%) were CIEP positive and all were males. Although none of the eight females that were tested by CIEP were sero-positive, the difference between the two sexes was not significant (χ^2^ = 2.8, *P* = 0.102), because of the small sample size. The results of duplicate CIEP tests were the same in all 466 samples, showing 100% concordance and demonstrating the high reproducibility of this test.

### Comparison between the two PCR primers

Of the 114 animals that were PCR positive by either of the primer pairs, 70.2% were amplified by both, 25.4% by the P60 only, and 4.4% by P70 only, showing that the P60 was somewhat superior over P70 in detecting the virus. Percentages of successful PCR amplifications in mink by both primers, P60 only and P70 only were 75.4%, 22.8% and 1.8%, respectively. The corresponding figures were 76.7%, 20.9% and 2.3% in weasels, and 11.1%, 66.7% and 22.2% in raccoons, showing that while P70 had negligible effects on improving the likelihood of detecting AMDV DNA in mustelids, its effect in raccoons was considerable (22.2%). The simple Kappa coefficients between the results of the two primer pairs were 0.39 (95% CL: 0.16 to 0.63, *P* = 0.001), 0.66 (95% CL: 0.47 to 0.84, *P* = 0.011) and 0.16 (95% CL: -.18 to 0.49, *P* = 0.16) for the mink, weasels and raccoons, respectively. AMDV DNA was detected by both primers in the two PCR-positive bobcats, the only skunk and one of the two otters, while one of the otter DNA samples was amplified by P70 only, showing that the use of this primer increased the chance of detecting AMDV DNA in this species.

### DNA extraction methods

Viral DNA was detected in the spleen of 87.3% and 36.1% of the 64 mink by the 60F/60R primer pair using DNA extracted by the commercial kit and the high-salt method, respectively. PCR amplification was successful in 33.3% of the samples extracted by both methods, 54.0% by the commercial kit only and 3.2% by the high-salt method only. Although use of the commercial kit considerably improved detection of viral DNA, the difference was not statistically significant (χ^2^ = 0.5, *P* = 0.86).

### DNA volume

Three DNA volumes were used because viral DNA concentration obtained from the cell-free media was too low to be accurately measured by a spectrophotometer. The effect of DNA volume on PCR success was analyzed using samples with at least one PCR positive reaction by either primer pair. PCR amplification successes were 54.1% (95%CL: 49.2-58.9%, n = 405), 43.8% (95%CL: 38.9-48.7%, n = 404) and 35.5% (95%CL: 30.9-40.2%, n = 411) for 1.5, 2.5 and 3.5 μL of DNA per 15 μL PCR reaction volume, respectively. The 1.5 μL DNA volume resulted in significantly higher PCR amplification success than the 2.5 μL (χ^2^ = 146.3, *P* < 0.001) and 3.5 μL (χ^2^ = 108.4, *P* < 0.001), which were also different (χ^2^ = 225.9, *P* < 0.001). Only 30.9% of the reactions (95%CL: 26.3-35.5%) were amplified by the three DNA volumes, while 59.7% (95%CL: 54.8-64.7%) were amplified by at least one reaction within each primer, implying that detection of infected animals almost doubled by using three DNA volumes. The negative relationship between DNA volume and PCR amplification success was possibly the result of PCR inhibitors that were not removed by the DNA extraction kit.

### Distribution of AMDV across the province

Mink and weasels were trapped in eight and seven of the counties, respectively, and viral DNA was detected in both species in all of these counties (Figure 1 and Table 1). No mink was trapped in Halifax or Hants Counties. Differences in prevalence of AMDV among counties were not significant for mink (χ^2^ = 4.1, *P* = 0.85) or weasels (χ^2^ = 4.3, *P* = 0.63), but positive raccoons were detected only in three of the eight counties in which they were trapped (χ^2^ = 30.2, *P* < 0.01). Prevalence of positive mink was 100% (n = 2) in the eastern part of the province (Pictou), 90.6% (n = 32) in the central region (Cumberland, Colchester, Halifax, Hants, Kings) and 96.2% (n = 26) in the west (Annapolis, Lunenburg, Queens, Yarmouth) (χ^2^ = 0.9, *P* = 0.65). The prevalence of positive weasels was 71.8% (n = 39) in the central and 68.2% (n = 22) in the western part of the province (χ^2^ = 0.1, *P* = 0.77). Positive raccoons were detected only in the central part of the province (14.1%, n = 64).

## Discussion

In addition to the members of the *Mustelidae* family (mink, weasel, otter, fisher) which are expected to be susceptible to infection by the AMDV, other furbearing carnivores (coyote, fox, skunk, raccoon, bobcat) and rodents (beaver, muskrat, squirrel) were tested primarily because they share habitat, prey upon, or scavenge for the cadavers, of potentially infected individuals (17,18).

The prevalence of AMDV in wild American mink (93.7%) in this survey was much higher than previous reports in Ontario (Canada), 55.2% of 29 mink [4], 61.7% of 120 mink [2], 29.0% of 208 mink [5], in Newfoundland, 44.4% of 18 mink [1] and in NS, 78.6% of 14 mink [3]. In Europe, the prevalence varied by country, with 23% of 75 mink in France [6], 52% of 27 mink in England [9], 3.1% of 396 mink from mainland Denmark and 45.1% of 142 mink on Bornholm Island in the Baltic Sea [7]. In Spain, none of the serum samples from the 14 American mink tested by CIEP was positive, but viral DNA was detected by PCR in the two pooled tissues of the five mink that were tested [8].

The high prevalence of AMDV in free-ranging mink in the current survey was likely due to the method of detection employed, namely CIEP and PCR using DNA extraction by a commercial kit, two primer pairs and three DNA volumes. The results of this survey and the published information suggest that free-ranging American mink have high prevalence of antibodies or AMDV DNA in Canada and many other regions of the world where the virus is present in the wild, and the differences in the estimated prevalence are primarily due to sample size and detection techniques. It is also possible that the prevalence of AMDV in trapped mink and other furbearers in this study is somewhat biased because such individuals may be clinically affected with reduced ability to hunt and thus are attracted to bait in traps, but this hypothesis needs further study.

This is the first report which demonstrated the presence of AMDV in the short-tailed weasel. The only report on the susceptibility of weasels to AMDV indicates that one of four weasels inoculated with AMDV became CIEP positive, but did not have an elevated level of γ-globulin or clinical signs [19]. The high rate of weasels carrying AMDV in the present survey (70.5%) is alarming and poses a considerable risk to the captive mink population, as well as to other wildlife, because of weasels’ wide range of habitat and the fact that they are preyed upon by large carnivores. The small size of weasels and their ability to easily climb trees and fences, and thus entering mink sheds in search of food, would make this species a prime biological vector of transmission of AMDV to ranched mink.

North American river otters had the 4^th^ highest rate of AMDV prevalence (18.2%) amongst the 12 species studied. The occurrence of natural infection by AMDV in North American river otters has not been studied, and a detectable level of antibody to AMDV by CIEP, an elevated level of γ-globulin or clinical signs were not observed when a single otter was experimentally inoculated with AMDV [19]. In Spain, AMDV was detected by PCR in the carcass of one dead Eurasian otter (*Lutra lutra*), a close relative of North American river otter [8], and clinical and pathological evidence showed that the death of a sick Eurasian otter in Britain was because of infection with this virus [14]. Our findings imply that North American river otters could be a reservoir of AMDV in the wild, but there is no information on the development of clinical disease in infected animals. Viral transmission between otters, free-ranging American mink and weasels is possible because otters sometimes prey on small mammals [17]. Further investigation is needed on a larger number of otters to provide a more accurate assessment of AMDV prevalence in this species in North America.

The finding that fishers did not carry AMDV is consistent with the previous report, which showed no CIEP positive individuals among 47 fishers tested in Ontario [4]. Two fishers that were experimentally inoculated with high doses of AMDV showed increased antibody titers over three years when tested by CIEP, but none showed elevated levels of γ-globulin or AMDV-induced clinical disease [19]. Natural exposure of fishers to AMDV is conceivable because they prey on weasels [18]. The information currently available suggests that either the fisher is one of the few members of the *Mustelidae* family which is not susceptible to natural infection by AMDV, or the prevalence of animals carrying the virus is very low in this species and larger sample sizes are needed for detection of infected animals. The small number of fishers tested in this survey (six) could be the reason that no infected individual was detected, and thus the results should not be considered as proof that fishers in NS are not infected with the AMDV.

The results of this survey and the presence of CIEP positive individuals in 65.3% of 196 striped skunks from Ontario [4], 77.1% of 48 breeder male skunks from a commercial operation in the USA [19], and 27.3% of 22 free-ranging skunks from South Dakota, USA [12], clearly show that this species is susceptible to natural infection by the AMDV. AMDV was detected by PCR in three sick striped skunks [10,13], and microscopic lesions indicative of AMDV infection were found in one of 177 skunk carcasses tested [20], implying that skunks can be infected and develop clinical signs of the disease. The results of experimental inoculation of striped skunks with the AMDV are not conclusive. No microscopic lesions was observed in experimentally inoculated striped skunks, and test mink were not infected when injected with organ homogenates prepared from skunks 80 days post-inoculation [4]. Increased γ-globulin levels (>15% of serum proteins), as well as renal and hepatic lymphocytic inflammatory changes were observed in striped skunks inoculated with this virus, but the lesions were different from those seen in AMDV infected mink and ferrets [19]. The increase in γ-globulin in the latter study could have been the result of other infections as elevated levels of γ-globulins (up to 29% of total serum proteins) were also observed in some CIEP-negative skunks from a commercial source [19]. Only eight skunks were tested in this survey and the estimated percentage of individuals carrying the virus (25%) should not be extrapolated to the entire skunk population in NS, but should be interpreted with caution. Striped skunks are widely distributed in NS [18], and thus could be an important reservoir for the AMDV in this province. Direct transmission of AMDV to mink, by skunks entering mink ranches which have biosecurity fences, may be slim. Skunks are occasionally hunted by coyotes, foxes, bobcats and fishers [18], and their cadavers may be consumed by smaller carnivores, causing the spread of AMDV in the wild.

In this survey, the low prevalence of raccoons carrying the virus or having antibodies (9.4%) is in agreement with the previous report which showed seroprevalence in 3.7% of 27 raccoons in Ontario [4]. Raccoons which are naturally infected by AMDV have been reported [12], and experimentally infected individuals produced antibodies to AMDV but showed no clinical signs of the infection. The diverse habitat and wide range of distribution of raccoons in NS, and their inquisitive nature, would make this species a high risk vector for infecting ranched mink, as was observed in the early 1990s in Utah, USA [12]. As scavengers, raccoons may consume carcasses of infected animals and could transmit the virus to larger carnivores which prey on this species, making them a potential vector for the spread of the virus in the wild.

This is the first study which reports the presence of AMDV in bobcats, and the results should not be surprising as the common genets (*Genetta genetta*), a member of the sub-order *Feliformia*, to which bobcats belong, was naturally infected and produced antibodies to AMDV [6]. The domestic cat (*Felis catus*), which belongs to the same subfamily as the bobcat (*Felinae*), showed antibodies to AMDV as early as day 7 post infection (pi), when experimentally inoculated with the Utah strain of the virus. Furthermore, since its organs contained infectious viruses 28 days pi, the cat was considered a potential AMDV reservoir [21]. Bobcats prey on small mammals, such as mink and weasels [17], thus becoming exposed to AMDV. Direct transmission of the virus from bobcats to ranched mink is not expected as they are solitary animals [17] and will not usually attempt to enter a mink shed.

Although coyotes and red foxes prey on small mammals, including mink and weasels [17,18], this survey suggested that they did not carry the virus. Two of 100 foxes tested in Ontario were CIEP positive [4]. Assuming the same rate of AMDV exposure in the fox population in NS, our sample size was too small to contain a positive animal. Blue (arctic) foxes (*Vulpes lagopus*), which belong to the same genus as the red fox, produced antibodies to AMDV when inoculated with the Utah strain of the virus, but they failed to support viral replication and were considered an unlikely reservoir of the virus [21]. Consistent with the results of the present survey, none of the five coyotes in the Ontario study were seropositive [4]. Sample sizes in the current experiment and the previous study [4] were too small to detect an infected individual and make a definite premise on the status of coyotes. After violet mink, raccoon dogs (Finn raccoons) (*Nyctereutes procyonoides*) were the most susceptible of the eight species that were inoculated with the Utah strain of AMDV (cat, dog, ferret, blue fox, mouse, rabbits) [21]. Raccoon dogs produced high antibody titers against AMDV, supported a considerable level of viral replication, showed some lesions in their kidneys that were similar to those observed in early stages of AMDV infection in the mink, and carried infectious viruses four weeks pi. The responses of raccoon dogs to AMDV inoculation were stronger than those in ferrets, a species that is known to be susceptible to infection by AMDV.

Experimentally inoculated dogs (*Canis lupus*), another member of the genus *Canis*, produced antibodies against AMDV and supported viral replication. However, the evidence that all inoculated individuals harbored infectious viral particles four weeks pi was inconclusive, and it was concluded that dogs may be a reservoir host for the virus [21]. These observations imply that some members of the *Canidae* family, to which the raccoon dog, domestic dog, coyote, red fox and arctic fox belong, may be susceptible to AMDV infection. The information available to date is not adequate to state, with any degree of certainty, whether or not red foxes and coyotes are potential reservoirs for AMDV.

None of the three species of rodents; muskrat, American beaver and red squirrel that were sampled in this survey carried AMDV. The only study on the natural infection of rodents by AMDV showed that none of the seven ground hogs (woodchucks, *Marmota monax*) in Ontario were seropositive [4]. The Syrian hamsters (*Mesocricetus auratus*) did not support viral replication when experimentally inoculated with AMDV [19]. The available information suggests that rodents do not become naturally infected with AMDV and are not risk factors for viral transmission. Yet, it has been shown that mice (*Mus musculus*) which were experimentally inoculated with the Utah strain of the AMDV, produced low levels of antibodies to AMDV by two weeks pi, and their tissue homogenates contained infectious virus particles [21]. It was concluded that mice may be considered a risk factor for AMDV transmission. The role that mice, and other small rodents such as squirrels, could play in AMDV transmission is particularly important because they frequently roam in and around mink ranches, and can easily penetrate most biosecurity fences. Further investigation of a large number of mice and squirrels from the vicinity of AMDV-infected ranches is required for a more accurate assessment of the potential risk from these species.

The peak of viral replication at around 10 days pi in mink is typically followed by a decrease in viral replication of up to several orders of magnitude over the succeeding few weeks, and the virus is sequestered in some individuals [21-25], resulting in negative PCR tests due to low viral copy number. The limited available information [4,12,21] suggests that viral replication in non-mink species is transient, and PCR only determines temporal presence of the virus. In addition, considerable sequence variation exists among the AMDV strains which circulate in captive mink [26], free-ranging American mink and other wildlife [8,10,12,13]**]**. Differences between the PCR primer sequences and the target genome could cause PCR amplification failure in animals that carry the virus. The observation that the two primer pairs (60F/60R and 70F/70R) were comparable in detecting the virus in the two mustelids (mink and weasels), while 70F/70R primers increased the detection of viral DNA in raccoons by 22.2% over the 60F/60R primers indicates the effect of sequence variation on PCR success rate among viruses circulating in these species. The results suggest that using a larger number of primers in species with unknown virus sequences is necessary to improve the chance of detecting infected animals by PCR.

The CIEP test on serum or plasma is most commonly used for detecting anti-AMDV antibodies in the mink. Production of antibodies to AMDV persists in the mink long after the cessation of viral replication, possibly as a result of virus sequestration [21], making CIEP an accurate measure of mink exposure to the virus. False negative CIEP tests are the result of low antibody titers during the early stages of exposure to the virus [27,28], and testing mink that are genetically prone to produce low antibody titers [2,22,29], can cause false negative CIEP test results. The limited published information suggests that antibody titer in wildlife is generally lower than that in the American mink [12,19,21,30], resulting in a lower sensitivity of the CIEP test in non-mink species. Differences within and among wildlife for the kinetics of antibody production and viral replication, as well as differences in the sequence of AMDV and viral sequestration have caused discrepancies between the results of PCR and CIEP tests in free-ranging American mink [7], skunks [10,12] and raccoons [12]. A combination of PCR and CIEP would improve the likelihood of detecting exposure of wildlife to AMDV.

Antibody titers in organs and tissues relative to that in the blood are not known. A preliminary study has suggested that antibody titer in the mink spleen homogenate is approximately 1.5 log (32 fold) lower than that in fresh plasma (unpublished data), which may explain the absence of any CIEP-positive weasels and the low percentage of CIEP positive mink (25%). The results imply that CIEP on tissue homogenate is not a reliable test and should not be used as the only test for detecting AMDV exposure in any species. The CIEP test on spleen homogenate, however, resulted in the detection of additional three positive mink and one positive skunk compared to PCR alone. These cases were either due to an absence of viral replication in individuals with massive amounts of circulating antibodies or the result of differences in sequences of the viruses carried by these animals.

More than 80% of mink ranches in NS are located in the western part of the province, and a considerable number of these ranches are infected with AMDV, while those in the central part of the province are mostly free of the virus [31]. Similarities among the counties and regions for the proportion of infected free-ranging mink and weasels are an indication that infected captive mink ranches are not directly related to the prevalence of free-ranging animals carrying the virus. The results suggest that either the virus has been indigenous to wild mink and weasels in NS, or it originated from captive mink several decades ago. The high prevalence of AMDV in free-ranging mink and weasels in the central region (Colchester, Cumberland, Hants, Kings), with a high proportion of AMDV-free ranches [31] is in agreement with the previous study on feral mink [3], and demonstrates that with proper biosecurity measures, AMDV-free ranches can be maintained in areas where the wildlife populations carry the virus. The small number of animals trapped in Yarmouth County, which has a large number of mink ranches, prevents a detailed analysis of the relationship between AMDV prevalence in wildlife and captive mink.

Similarity of the two sexes for the proportion of mink, weasels and raccoons carrying AMDV is in agreement with other reports on free-ranging mink [5,9] and six species of free-ranging carnivores [6]. The observation that 85.7% of the mink, 89.5% of weasels and 67.1% of raccoons whose genders were recorded were males in this survey is consistent with another report [5] showing that 63.9% of 208 free-ranging mink trapped in Ontario were males. The reasons for the higher proportion of males trapped is not clear, but harvesting fewer females would have a positive effect on the survival of these species.

Although AMDV was first reported in captive American mink [32], the origin of the virus in wildlife is uncertain. There is a wide spectrum of parvoviruses which infect different animal species [33]. Therefore, it is logical to assume that some strains of AMDV or AMDV-like viruses have been circulating in the populations of wild American mink and other wildlife in North America, long before the start of fur animal farming. It would be presumptuous to assume that the AMDV appeared spontaneously in captive mink, and that the source of AMDV in free-ranging North American mink is the captive mink [5]. Mink farming has possibly played a role in expanding the range of contamination and increasing its prevalence through the escape or release of infected animals and disposal of contaminated cadavers and waste materials. The large number of infected captive mink, kept under conditions of high density, offers an opportunity for the rapid accumulation of new viral strains, as a result of the high mutation rate of the virus [34]. Thus, mink ranches could be a source of a continuous supply of new strains of AMDV to the wild, and some new isolates may be pathogenic for wildlife. The higher seroprevalence of AMDV in free-ranging mink in areas closer to mink ranches in Ontario than in areas farther from ranches was considered proof that mink ranches act as sources of AMDV transmission to the wild [5]. Although the spread of the virus from infected ranches to the wild cannot be underestimated, other factors may affect regional differences in the prevalence of animals carrying AMDV. Free-ranging animals in search of food, for example, may be more concentrated around mink ranches, which give rise to higher rates of horizontal transmission of the virus due to the proximity of animals to each other.

## Conclusions

Controlling AMDV in captive mink herds in NS is difficult because vast areas of the province are inhabited by wildlife that carry the virus and roam around mink ranches in search of food and may come in direct contact with captive animals if they pass through perimeter fences. Although implementation of rigid biosecurity systems would protect clean ranches, several biological vectors, such as insects [35] and birds may transfer the virus from cadavers or feces of infected free-ranging animals to the captive mink. The escape or release of captive mink to the wild may cause the spread of viral strains that could be pathogenic for wildlife. There is a need to understand the movement of the virus between captive mink and wild animal populations, and to document the role of viral strains in causing health issues for different wild animal species.

## Competing interests

The author declares that he has no competing interest.
